# Examining the contribution of cell wall polysaccharides to the mechanical properties of apple parenchyma tissue using exogenous enzymes

**DOI:** 10.1093/jxb/erx329

**Published:** 2017-09-23

**Authors:** Pauline Videcoq, Adelin Barbacci, Carole Assor, Vincent Magnenet, Olivier Arnould, Sophie Le Gall, Marc Lahaye

**Affiliations:** 1 INRA, UR1268 Biopolymères Interactions et Assemblages, Nantes, France; 2 Université de Strasbourg, UMR 7357 Laboratoire des Sciences de l’Ingénieur, de l’Informatique et de l’Imagerie (ICube), CNRS, Illkirch, France; 3 Université de Montpellier, LMGC, CNRS, Montpellier, France

**Keywords:** Apple, biomechanics, cell wall, enzyme, mechanical properties, model, parenchyma

## Abstract

The viscoelastic mechanical properties of water-rich plant tissues are fundamental for many aspects of organ physiology and plant functioning. These properties are determined partly by the water in cellular vacuole and partly by the mechanical properties of the cell wall, the latter varying according to the composition and organization of its polysaccharides. In this study, relationships between the viscoelastic properties of apple cortex parenchyma tissue and cell wall pectin, hemicelluloses, and cellulose structures were studied by infusing the tissue with selected sets of purified enzymes in a controlled osmoticum. The results showed that tissue elasticity and viscosity were related, and controlled to variable extents by all the targeted polysaccharides. Among them, pectic homogalacturonan domains, crystalline cellulose, and fucosylated xyloglucan were revealed as being of prime importance in determining the viscoelastic mechanical properties of apple cortex tissue.

## Teaser

The impact of enzymatic degradation of cell walls on the viscoelasticity of apple cortex tissue reveals that methylated homogalacturonan, crystalline cellulose, and xyloglucan branching are key structures in determining cell wall mechanical properties.

## Introduction

Many aspects of plant functioning are linked to the mechanical properties of plant tissues. Cell walls and compartmentalization of water contribute to creating turgor pressure, leading to the formation of a hydrostatic skeleton that is fundamentally responsible for the viscoelastic mechanical properties of water-rich tissues, such as those found in fleshy fruit (Passioura, 1994). Polysaccharides, together with small amounts of structural proteins, form the basis of cell wall mechanical properties (Albersheim *et al.*, 2011) but elucidating their precise role remains a challenge. Focusing on polysaccharides, the cell wall generally comprises pectin with varying amounts of methyl-esterified homogalacturonan (HG), ramified rhamnogalacturonan I (RGI) and II (RGII) structural domains (Atmodjo *et al.*, 2013). Walls are also made of hemicelluloses with xyloglucan (XyG), mannan, and xylan families (Scheller and Ulvskov, 2010) and cellulose. Much has been learned about the chemistry, physicochemistry, and interactions of isolated cell wall polysaccharides (Albersheim *et al.*, 2011). Glucan chains in cellulose form partially crystalline structures via hydrogen bonds, while pectins assemble through ionic interactions of HG and through borate ester-mediated dimerization of RGII structural domains. Galactan and arabinan side-chains of pectic RGI domains bind to cellulose through hydrogen bonds (Zykwinska *et al.*, 2005; Lin *et al.*, 2015). Among hemicelluloses, xyloglucan partially hydrogen-binds to cellulose and is, to a minor degree, intertwined within the microfibrils (Park and Cosgrove, 2015). It is also able to self-assemble (Shirakawa *et al.*, 1998) but fine variations in its complex structural building blocks (Tuomivaara *et al.*, 2015) probably modulate its interactions (Hanus and Mazeau, 2006; Zhao *et al.*, 2014). Mannan and xylan can also self-assemble and bind to cellulose through hydrogen bonds depending on the presence of substituents (acetyl esters, side-chains) and/or in chain-substitution of mannose by glucose residues (Nieduszynski and Marchessault, 1972; Chanzy *et al.*, 1984; Vian *et al.*, 1994; Whitney *et al.*, 1998; Richardson *et al.*, 1999; Penroj *et al.*, 2005; Kabel *et al.*, 2007). All these matrix polysaccharides that embed and interact with cellulose can also be partly linked to each other covalently (Nakamura *et al.*, 2002; Popper and Fry, 2008; Prakash *et al.*, 2012; Tan *et al.*, 2013; Ralet *et al.*, 2016).

Plant mutants (Ulvskov *et al.*, 2005; Burgert and Dunlop, 2011; Ruiz-May and Rose, 2013) and cell wall analogues composed of various cell wall polysaccharides incorporated in bacterial cellulose (Chanliaud *et al.*, 2004; Whitney *et al.*, 2006; Cybulska *et al.*, 2010; Gu and Catchmark, 2013; Lin *et al.*, 2016) are particularly helpful in deciphering the contributions of cell wall polysaccharides to tissue mechanical properties. They have pointed to key roles of xyloglucan and pectin, and of pectin-degrading enzymes (endopolygalacturonase, pectin lyase), xyloglucan-remodeling enzymes (xyloglucan endotransglucosylase/hydrolase, XTH), and expansin proteins that regulate xyloglucan–cellulose interactions (Brummell *et al.*, 1999; Jiménez-Bermúdez *et al.*, 2002; Chanliaud *et al.*, 2004; Cantu *et al.*, 2008; Quesada *et al.*, 2009; Burgert and Dunlop, 2011; Atkinson *et al.*, 2012; Miedes *et al.*, 2013; Lin *et al.*, 2016; Minoia *et al.*, 2016). These observations have informed conceptual representations of cell wall macromolecular organization and functions (reviewed in Cosgrove, 2003), of which the most recent one proposes a scaffold of cellulose microfibrils embedded in pectin with a matrix of hemicelluloses keeping the microfibrils apart (Park and Cosgrove, 2015). According to this representation, minute amounts of XyG in strong interaction with cellulose will form biomechanical ‘hotspots’ that control cell wall creep. Furthermore, interactions of both pectin and hemicelluloses with cellulose will also contribute to the wall load-bearing.

To further examine the viscoelastic mechanical contribution of cell wall polysaccharides, we modulated cellulose, hemicellulose, and pectin structures by vacuum-infiltration of selected enzymes in the porous parenchyma tissue of the cortex of apple fruit. The elastic and viscous properties of turgor-controlled tissue were measured to assess the impact of enzymatic modifications of cell wall polysaccharides on the tissue’s mechanical properties. To determine the role of the affected polysaccharides on the tissue’s mechanical properties a model was developed that allowed compensating disparities between enzyme affinities and the variability of the fruit parenchyma tissue.

## Materials and methods

### Plants

*Malus* ×*domestica* Granny Smith (Gr) and Golden delicious (Go) were purchased from a local producer (Pommeraie Nantaise, Nantes, France) approximately 15 d after harvest and stored at 4 °C until use. Apples were selected at random and kept at room temperature (around 17 °C) overnight prior to experiments.

### Chemicals

Morpholino-ethanosulfonic acid, L(+)-ascorbic acid, calcium chloride, and D-mannitol were obtained from Sigma-Aldrich (Fluka, Riedel de Haen; St Quentin Fallavier, France). Dimethylsulfoxyde was obtained from VWR Chemicals (BDH-Prolabo; Fontenay-sous-Bois, France).

### Enzymes

Enzymes were obtained from Megazyme (Bray, Ireland), Sigma-Aldrich, or were prepared in the laboratory. Their activities (1 unit = 1 µmol substrate released per minute hydrolysis) were checked in the laboratory before use. The different enzymes used, their coding, source, and activities are given in Table 1. Preliminary experiments were carried out to determine the appropriate enzyme concentrations that would give observable effects on the mechanical properties of infused apple tissue after 5 h of incubation and yet would avoid complete destruction of the sample. All enzymes were used in an infusion buffer (see below). When enzymes were combined, the same total amounts of activity were added as when the enzymes were used alone.

**Table 1.** List of enzymes, code used, source, and activity used in assays

Enzyme	Code	Source	Specific activity used (U ml^–1^)
α-Arabinofuranosidase	af	Megazyme; *Bifidobacterium adolescentis* E-AFAM2	8
α-Fucosidase	afs	Megazyme; *Thermotoga maritima*, E-FUCTM	0.125
α-Arabinanase	an	Megazyme; *Aspergillus niger*, E-ARAB	2.5
β-Galactosidase	bgs	Megazyme; *Aspergillus niger*, E-BGLAN	40
Cellobiohydrolase	ch	Megazyme; *Trichoderma longibrachiatum* E-CBHI	0.01
Cellulase	can	Megazyme; *Aspergillus niger*, E-CELAN	20
	ctl	Megazyme; *Trichoderma longibrachiatum*, E-CELTR	20
Galactanase	gn	Megazyme; *Aspergillus niger*, E-GALN	10
Endopolygalacturonase	pg	Megazyme; *Aspergillus aculeatus*, E- PGALUSP	60
Pectin lyase	pl	Peclyve: *Aspergillus niger* (Ralet *et al.*, 2012)	0.0025
Xyloglucanase	xn	Megazyme; *Paenibacillus* sp., E-XGP	10
Pectin methyl esterase	pme	Sigma; Orange peel	3.5

### Chemical analysis

The sugar composition of apple parenchyma was determined at the end of viscoelastic measures for samples infused with an isotonic solution and no exogenous enzymes. Freeze-dried apple parenchyma cell walls were prepared as alcohol-insoluble material (AIM) as reported by Winisdorffer *et al.* (2015). AIMs were dried at 40 °C overnight under vacuum over P_2_O_5_ before grinding and weighing.

Identification and quantification of neutral cell wall sugars were performed by gas–liquid chromatography after a two-step degradation in sulphuric acid as described by Winisdorffer *et al.* (2015). Sugars were analysed by gas chromatography after conversion to alditol acetates as described by Winisdorffer *et al.* (2015). Uronic acids in acid hydrolysates were quantified using the meta-hydroxydiphenyl colorimetric acid method (Blumenkrantz and Asboe-Hansen, 1973).

### Experimental design and test sets

A total of 17 enzymes or combinations were used to study the relationships between enzyme hydrolysis and the mechanical properties the cortex parenchyma of the Go and Gr varieties. Each test was performed on a randomized date over the 106 d of the experimental period on four different fruits of each variety. A slice of cortex parenchyma tissue about 1 cm thick was taken at the equator of the fruit. In each slice, nine cylinders (mean size 1 cm height × 0.8 cm diameter) were sampled at 5 mm from the epidermis as described previously (Gálvez-López *et al.*, 2012). The cylinders were then infused with an isotonic solution with or without exogenous enzymes and kept immersed during the entire duration of the experiment. Elastic and viscous behaviors, described by the storage modulus *E′* and by damping, tan(*δ*), respectively, were measured by Dynamic Mechanical Analysis (DMA; see below) performed every hour on each sample over the course of 5 h. For each combination of enzyme and apple variety, the effect of enzyme hydrolysis was assessed by comparison between a pool of samples infused with the control buffer that did not contain any enzymes (4 apples × 4 samples/apple = 16 samples every hour) and a pool of samples infused with enzymes (4 apples × 5 samples/apple = 20 samples every hour).

### Isotonic buffer

The mechanical properties of parenchyma tissue are functions, at the first order, of cell wall mechanical properties and the water content of the tissue (Gibson *et al.*, 2010). To specifically test the effects of cell wall modifications on tissue mechanical properties, an isotonic buffer was designed to avoid changes in water content during the experiment. The composition of the isotonic buffer was adjusted in preliminary experiments to keep the elasticity constant over 5 h. It was based on mannitol (0.72 M for Gr and 0.67 M for Go) at concentrations close to those reported in the literature (Oey *et al.*, 2007). pH 6 was maintained by morpholino-ethanosulfonic acid (2 mM). A small amount of dimethylsulfoxyde (2.8 mM) was added to foster diffusion of the buffer in the sample, ascorbic acid (5.6 mM) was added to limit endogenous oxidation, and calcium chloride (0.25 mM) was added to support optimal enzyme activity.

### Vacuum-infiltration of samples

All samples were infused under partial vacuum (50 mbar) for 30 s, which was the time previously reported to achieve total impregnation of a 1-cm^3^ sample (Guillemin *et al.*, 2006). The nine cylindrical tissue samples were split into two groups: five samples were infused with the test enzyme or enzyme combination (see Table 1 for abbreviations used) in the isotonic buffer, and four were infused with buffer only to evaluate the effect of endogenous enzymes (termed ‘endo’ hereafter). Once infused, samples were kept immersed in the solution at room temperature (17 °C) during the whole experiment (5 h). Microscope observations were performed in preliminary tests to check for the absence of cell plasmolysis or bursting (see Supplementary Fig. S1 at *JXB* online).

### Mechanical assay

The rheological behavior of plant tissues is viscoelastic for small mechanical loads within the reversible compression range. Elasticity refers to the tissue resisting deformation without any damage. The elastic response to a mechanical load is instantaneous and reversible. Elastic modulus is defined as the ratio between stresses and strains for a given loading direction. Viscosity refers to the stress or strain relaxation. The viscous response to mechanical load is delayed in time.

The viscoelastic behavior of the cell walls was assessed by DMA using a Bose ElectroForce 3100 (Bose Corporation, Eden Prairie, MI, USA). The linear region of viscoelasticity of apple parenchyma was found for strains lower than 1% (Menard, 2008). The poroelastic characteristic time of water diffusion in apple samples is greater than 10 s (Dumais and Forterre, 2012). Hence, the compressive strain was applied with a sinusoid of 1 Hz frequency to avoid water flux (measurement at constant volume) and 0.4% amplitude at around 0.5% of mean strain to remain within the linear viscoelastic range. The duration of the test was 20 s, i.e. 20 cycles of compression and release. The complex elastic modulus (termed E*) is defined as the ratio between the maximum stress value, caused by the strain, and the maximum strain value. Viscosity is relative to the phase angle (termed *δ*), which is the delay between the applied strain and the measured stress. On each sample, the test was performed every hour, six times. The exact time of each assay was recorded. The temperature of the room was constant at around 17 °C. The storage modulus [E′=E*cos(*δ*)] and damping [tan(*δ*)] represent the energy stored (elasticity) and dissipated (viscosity) by the material. *E′* is almost equal to the usual elastic Young’s modulus for a material with low damping (which is the case here; see below).

### Data processing

The effects of enzymes were assessed using Welch’s unequal variances *t*-test on the variation of *E′* over the 5 h of the experiment between samples infused with exogenous enzymes and control samples infused with isotonic buffer only.

### Statistical analysis and regressions

The errors in *E′* and tan(*δ*) were equally taken into account, and there was no reason to minimize the sum of squares in a preferential direction by using the usual least squares. Orthogonal regressions were computed by minimizing the sum of squares in two directions by least-rectangles regression (Dagnelie, 2007).

Student’s/Welch’s tests and least-rectangles regressions were performed using R software and libraries (R Development Core Team, 2013; Wickham, 2009).

A model describing *E′* and tan(*δ*) during the hydrolysis of polysaccharides was developed in a statistical mechanics framework for low *δ* values and systems close to the thermodynamical equilibrium. This framework has already been used to model mechanical properties of polymers (Mrabet *et al.*, 2005), cell growth (Barbacci *et al.*, 2013), and the mechanical properties of Ca^2+^-alginate gels (Magnenet *et al.*, 2012). The model showed that in the most general case, storage modulus and damping are functions of parameters describing coupling between mechanical, thermal, and chemical energies and the frequency of mechanical stress (Supplementary Data, Modeling, equations 9 and 11). General equations were simplified under the assumptions of low damping and the state of the sample being close to mechanical equilibrium. These simplified equations were designed to relate storage modulus variation to damping variation (Supplementary Data, Modeling, equation 17) and to the effect of hydrolysis over time (equation 18). Enzyme activities were considered constant over time. Detailed mathematical developments are provided in Supplementary Data, Modeling.

## Results and discussion

### Mechanical properties and cell wall polysaccharide composition of apple cortex tissue

**Fig. 1.**
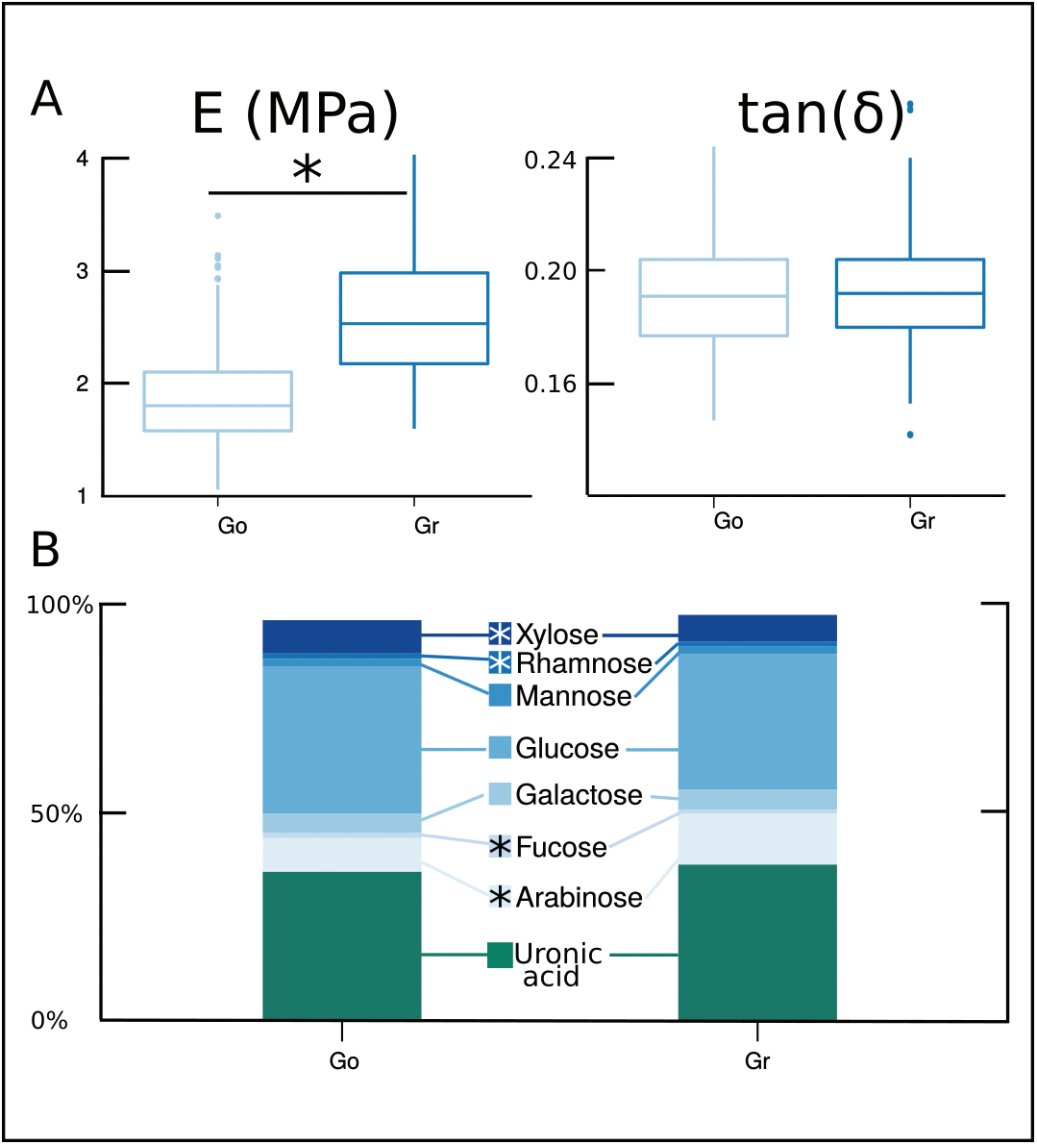
Descriptive statistics of storage modulus (*E′*) and damping [tan(*δ*)] (A), and sugar composition (B) of Golden Delicious (Go) and Granny Smith (Gr) apple varieties. * Significantly different (*P*<0.05).

The cortical tissue of ripe Granny Smith (Gr) apples had a higher storage modulus than that of Golden delicious (Go) apples (Gr: *E′*=2.64 MPa; Go: *E’*=1.87 MPa; *P*<005) but similar damping values [tan(*δ*)=0.19, *P*=0.65] (Fig. 1A). These values were within the range of previously published data (Van Woensel and De Baerdemaeker, 1983; Abbott and Lu, 1996; Grotte *et al.*, 2002; Cen *et al.*, 2013).

The cell wall polysaccharides of the cortex of Gr and Go (Fig. 1B) were composed of uronic acid (~39% dry weight of AIM) and glucose (~35%) as the major component sugars, followed by arabinose (~11%), xylose (~8%), galactose (~4%), and low amounts of mannose (~1%), rhamnose (~1%), and fucose (~1%). This composition was typical of apple (Galvez-Lopez *et al.*, 2011, and references herein). Small significant differences between varieties for arabinose (4% higher for Gr, *P*<0.05), xylose (<1%, but higher for Go, *P*<0.05), rhamnose (<1%, but higher for Go, *P*<0.05), and for fucose (<1%, but higher for Go, *P*<0.05) may have reflected developmental and genetic differences.

Given that the mechanical properties of the Go and Gr cortex tissues were measured at constant water content, the difference in elasticity observed between the two varieties did not result from major differences in polysaccharide composition, but probably from small structural variations affecting their interactions. For example, the higher amount of arabinose in Gr has previously been correlated with higher elasticity (Winisdorffer *et al.*, 2015).

### Infused enzymes affect tissue viscoelastic properties

**Fig. 2.**
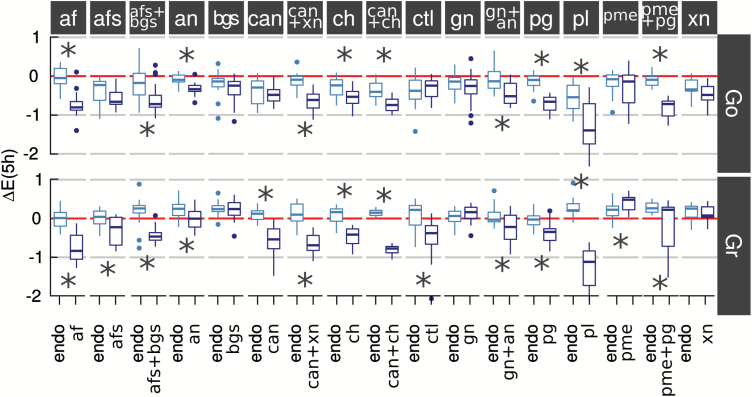
Effects of endogenous (endo) and exogenous enzymes on the storage modulus of Golden Delicious (Go) and Granny Smith (Gr) apple varieties after 5 h incubation in the reaction medium. Enzyme codes are given in Table 1. * Significanty different (*P*<0.05). Cellulase (can and ctl), pectin methylesterase (pme), and α-fucosidase (afs) did not affect the modulus of Go parenchyma tissue. Xyloglucanase (xn) did not modify the modulus of Gr. β-galactosidase (bgs) did not affect either Go or Gr. All other enzymes and combinations significantly affected the mechanical properties of the cortex parenchyma tissue in both Go and Gr.

Apple ripening and associated softening involve cell wall deconstruction by complex enzymes and consortia of other proteins (Brummell and Harpster, 2001; Goulao *et al.*, 2007; Dheilly *et al.*, 2016). An extensive examination of the endogenous proteins and enzymes of the Mondial Gala apple variety by Goulao *et al.* (2007) gives a good picture of the evolution of the relative activities over the course of fruit development and softening of (exo)polygalacturonase, pectin methylesterase, pectate lyase, β-galactosidase, α-L-arabinofuranosidase, endo-1,4-β-glucanase, xyloglucan endotransglycosylase, and expansin. However, most studies on apple endogenous enzymes have focused on polygalacturonase (pg), galactosidase (bgs), arabinofuranosidase (afs), and pectin methylesterase (pme) activities, which range from 0.06 to 23.0 (pg), 0.7 to 3.5 (bgs), 0.06 to 4.2 (afs), and 26 to 40 (pme) nmol g^–1^ FW min^–1^ depending on the variety and the storage conditions of the harvested fruit (Goulao and Oliveira, 2008; Wei *et al.*, 2010; Ng *et al.*, 2015; Gwanpua *et al.*, 2016). Infiltration of exogeneous enzymes was used to complement or to extend the action of cell wall-acting endogeneous enzymes in order to assess the impact of specific structural modifications of polysaccharides on the viscoelasticity of apple cortex tissue. Enzyme infusion is a process used to modify fruit to a desired texture, such as pme for altering the firmness of apple pieces (Guillemin *et al.*, 2006). Assuming a homogeneous distribution of the infused enzymes in the samples, the added activities were expected to be in large excess (µmol g^–1^ FW min^–1^) relative to normal endogenous ones that are measured in the nmol range (see above). Most of the infused enzymes in Go and Gr significantly affected *E′* after 5 h incubation (Fig. 2), and thus most of them contributed in the cleavage and re-organization of cell wall polysaccharides in the ripe fruit. However, β-galactosidase (bgs, *P=*0.143 for Go, *P=*0.776 for Gr), β-1,4-galactanase (gn, *P=*0.246 for Go, *P=*0.194 for Gr), and xyloglucanase (xn, *P=*0.118 for Go, *P=*0.593 for Gr) did not affect *E′* in spite of their high ability to hydrolyse substrate (enzymes activities are provided in Table 1). The lack of effect of β-galactosidase and β-galactanase on the mechanical properties of apple tissue was probably related to the important metabolism of galactose during fruit development and ripening, and particularly that affecting pectic rhamnogalacturonan I (RGI) galactan side-chains (Brummell and Harpster, 2001; Peña and Carpita, 2004; Ng *et al.*, 2015), which are thought to contribute to the cell wall mechanical properties by forming hydrogen bonds with cellulose (Zykwinska *et al.*, 2005; Lin *et al.*, 2015). The absence of an impact of the *Paenibacillus* xyloglucanase, which belongs to the glycoside hydrolase family 5 (GH5), is in line with results from cucumber and Arabidopsis hypocotyls that showed GH12 enzymes active on both cellulose and xyloglucan are required to cleave biomechanical ‘hotspots’ and allow creep to occur (Park and Cosgrove, 2012). Cellulases from *Trichoderma longibrachiatum* (ctl, GH5) and from *Aspergillus niger* (can, GH12) with, respectively, low and very low specific activities on xyloglucan, had no effect on the viscoelastic properties of Go (ctl, *P=*0.512; can, *P=*0.377) but affected those of Gr. Boosting *A. niger* cellulase with xyloglucanase (can+xn) had limited effects on the reduction of the storage modulus of both varieties. The contrasting responses of the mechanical properties of Go and Gr following cellulase treatment may have arisen from different substrate characteristics and/or enzyme inhibition. In a similar way, Go was also distinguished from Gr with regard to pectin methylesterase, since the *E′* value of Go was not significantly affected by pme (*P=*0.310) while it increased for Gr (*P*<0.05). Pectin methylesterase promotes the dimerization of HG mediated by Ca^2+^ (Videcoq *et al.*, 2011) and as a consequence contributes to strengthening cell walls and tissues (Guillemin *et al.*, 2006). The pme activity may have been differently modulated in the two varieties because it is sensitive to the distribution of non-esterified uronic acids, to calcium concentration (Videcoq *et al.*, 2011), and to cell wall pme inhibitors (Jolie *et al.*, 2010).

The effect of infused enzymes on the storage modulus may not always be directly due to cleavage of the polysaccharide structure but could also be indirect by making accessible polysaccharide moieties that promote endogenous enzyme activities, such as by creating xyloglucan side-chain structures with greater binding to XTH (Maris *et al.*, 2011), or via active oligosaccharides triggering chemioperceptive pathways that lead to expression of endogenous enzymes (Ryan and Farmer, 1991).

Sets of exogenous enzymes that led to limited *E′* variations (Go: afs, bgs, can, ctl, gn, pme, xn; Gr: bgs, gn, xn) were removed from the data used in the following discussion.

**Fig. 3.**
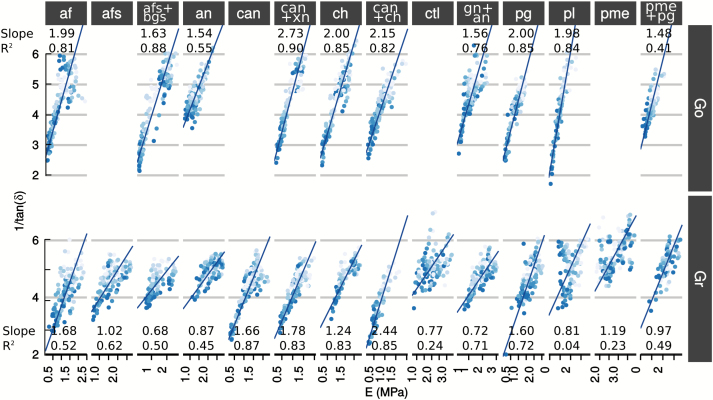
Linear relationships between 1/*E′* and damping [tan(*δ*)] for Golden Delicious (Go) and Granny Smith (Gr) apple varieties. The shading of the symbols in each graph indicates the time of sampling, from 0 h (dark symbols) to 5 h (light symbols). For each graph the values of the slope and Pearson’s *R*^2^ re stated.

During the 5-h experiments, damping [tan(*δ*)] appeared to be linearly related to the storage modulus, *E′* (Fig. 3, Supplementary Table S1). For small damping values (mean values were 0.20 rad for Gr and 0.22 rad for Go), damping scaled with the inverse of storage modulus (Supplementary Data, Modeling, equation 12):

$$\text{tan}\left(\delta \right)\propto \frac{1}{E´}$$ (1)

This relationship was fitted for each combination of enzyme and variety and was confirmed to be linear (Fig. 3, Supplementary Table S1). Similar relationships have already been reported in wood (Brémaud *et al.*, 2011) and recently for apple cortex tissue (Winisdorffer *et al.*, 2015). In wood, elastic modulus and damping values were linked to the mean microfibril angle, which explained the relationship between the two parameters (Ono and Norimoto, 1983, 1985). However, in the case of the fruit cortex, the role played by the cell wall polysaccharides appears to be more intricate as in ripe apple, the organization of cell wall polysaccharides is anisotropic. Furthermore, no particular polysaccharide was specifically associated with elasticity or viscosity as the relationship between damping and elasticity formed a continuum in which the elastic modulus was converted to viscosity. Polysaccharide fragments released by enzyme hydrolysis did not contribute to the resistance of the cell wall (*E′*), which decreased with time. Instead, they may have created locally weak interactions with other cell wall polysaccharides with the consequence of increasing damping.

### Key polysaccharide structures affecting the mechanical properties of apple parenchyma

The impact of a given polysaccharide on the mechanical properties of apple cortex tissue will be related to how much its binding contributes to the overall strength of the cell wall structure. This in turn will depend on the mean energy per bond and the number of bonds per amount of the given polysaccharide. Thus, hydrolysis of minute amounts of a tightly bound polysaccharide will have a drastic impact on mechanical properties. The contribution of polysaccharide structures on the mechanical properties of apple parenchyma tissue can therefore be evaluated by the variation of elasticity caused by a fixed quantity of polysaccharide hydrolysed (i.e. as a function of the enzyme active units used). Such an evaluation allows the high-binding-energy structure of the cell wall to be identified.

To understand further the evolution of mechanical properties during enzyme hydrolysis, a model was developed within a statistical mechanics framework (Magnenet *et al.*, 2012; Barbacci *et al.*, 2013). This model assumes low damping values and a system that is close to mechanical equilibrium in isothermal conditions (see Supplementary Data, Modeling). The effects of the local ionic environment, pH variations, accessibility of substrates, and the heterogeneity of hydration that is known to modulate enzyme activities were not taken into account, and constant enzyme activities were assumed.

Under these assumptions, the variation of the storage modulus (*ΔE′*) was found to be proportional to the enzyme activity, $\dot{\xi }$, multiplied by the time between infusion and testing (*Δt*), i.e. proportional to the quantity of polysaccharides hydrolysed (*ξ*):

$$\Delta E'=E-E_{0}=K_{1}\dot{\xi }\Delta t$$ (2)

The relative variation of tan(*δ*) was:

$$\frac{\Delta tan\left(\delta \right)}{tan\left(\delta \right)}=K_{2}\dot{\xi }\Delta t$$ (3)

**Fig. 4.**
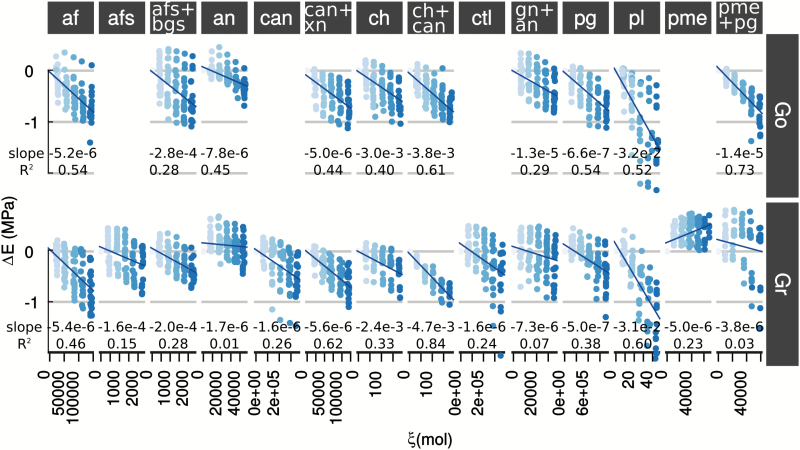
Linear relationships between variation of storage modulus (*ΔE′*) and number of hydrolysed polysaccharides (*ξ*) for Golden Delicious (Go) and Granny Smith (Gr) apple varieties. The shading of the symbols in each graph indicates the time of sampling, from 0 h (dark symbols) to 5 h (light symbols). For each graph the values of the slope and Pearson’s *R*^2^ are stated. The slopes correspond to the sensitivities of elasticity to hydrolysis. Thus, homogalacturonan domains hydrolysed by pl, crystalline cellulose hydrolysed by ch, and xyloglucan fucosylation appear to be key structural changes related to elasticity.

**Fig. 5.**
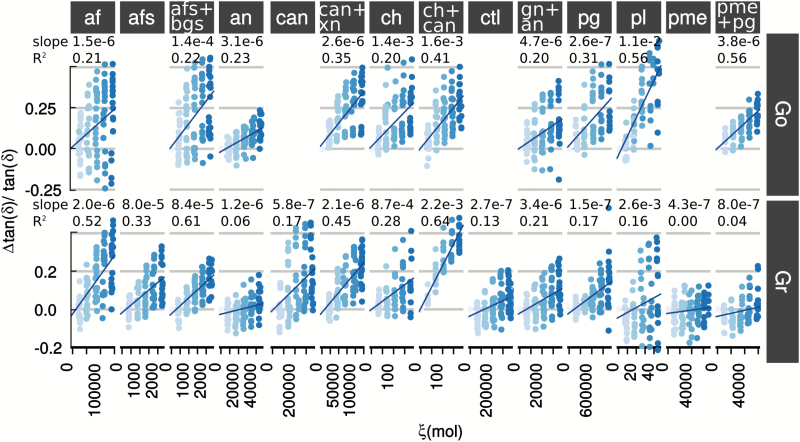
Linear relationships between variation of relative damping variation [*Δ*tan(*δ*)/tan(*δ*)] and number of hydrolysed polysaccharides (*ξ*) for Golden Delicious (Go) and Granny Smith (Gr) apple varieties. The shading of the symbols in each graph indicates the time of sampling, from 0 h (dark symbols) to 5 h (light symbols). For each graph, the values of the slope and Pearson’s *R*^2^ are stated. The slopes correspond to the sensitivities of relative viscosity variation to hydrolysis. Thus, homogalacturonan domains hydrolysed by pl, crystalline cellulose hydrolysed by ch, and xyloglucan fucosylation appear as key structural changes related to viscosity.

The linear form of Equations 2 and 3 was confirmed experimentally (Figs 4 and 5, Supplementary Tables S2 and S3) for all test sets. The relationship was a decreasing one except for pectin methylesterase in Gr due to cortex stiffening (increased *E′*) resulting from HG cross-linking by Ca^2+^ (see above).

The contribution of a given polysaccharide on the mechanical properties of the cortex tissue was assessed from the sensitivities of elastic variation (||*K*_1_||) and damping relative variation (*||K*_2_||) to exogenous enzymatic hydrolysis. Thus, ||*K*_1_|| and ||*K*_2_|| allowed the ranking of the contribution of each polysaccharide, and the higher the value of *||K*_1_|| and ||*K*_2_||, the more important is the involvement of the hydrolysed bond to mechanical properties.

The results clearly pointed to three key polysaccharide structures involved in the storage modulus and damping. These were methylesterified pectin (hydrolysed by pectin lyase, pl), crystalline cellulose (hydrolysed by cellobiohydrolase, ch), and the fucosylated xyloglucan side-chains (hydrolysed by α-fucosidase, afs) (Figs 4 and 5). The major impact of the cleaving of methylesterified HG by pectin lyase on the tissue viscoelastic properties is in keeping with its contribution to the load-bearing of the cell wall (Burgert and Dunlop, 2011; Lin *et al.*, 2016). In apple tissue, HG degradation also very probably affected cell–cell adhesion in digestion of the middle lamella (Jarvis *et al.*, 2003). In contrast, the mechanical properties were not particularly sensitive to the combination of pectin methylesterase and endopolygalacturonase that was expected to result in a similar structural change. The high enzyme activites required to impact on the properties, which were only seen in Go (Fig. 2), may have reflected the calcium-related regulation of pg and pme and/or the presence of pg and pme inhibitors (Jolie *et al.*, 2010). Apple pectin acetylesterification and substitution by xylose in xylogalacturonan was not thought to be the primary cause of this inhibition, as it would also have affected pectin lyase (Voragen *et al.*, 2009).

Unexpectedly, cellobiohydrolase (ch) with and without cellulase (can) was quite efficient in modulating viscoelastic properties. Cellobiohydrolase I from *Trichoderma viridae* hydrolyses free glucan end-chains processively on the hydrophobic faces of the crystalline region of cellulose (Liu *et al.*, 2011). With endo-glucanase, it acts synergistically to hydrolyse cellulose crystals (Béguin and Aubert, 1994). In the present case, adding *Aspergillus* cellulase, which cleaves the amorphous cellulose region and exposes glucan chains in the crystalline region, had no major impact on *E′*, and the endogeneous apple glucanase may have already played this role (Goulao *et al.*, 2007). As the hydrophobic faces of cellulose crystal are probably preferred sites for xyloglucan interactions (Zhao *et al.*, 2014), it would be of interest to test whether cellobiohydrolase activity on these sites alters the biomechanical ‘hotspots’ that are proposed to be targeted by expansin, which acts to disassemble the strong XyG/cellulose interactions (Park and Cosgrove, 2015). Identifying the effect of the *in muro* release of cellobiose on the nature of XET products would also be of interest since the disaccharide can potentially act as an acceptor (Baumann *et al.*, 2007) and may thus shorten the length of the xyloglucan molecules and consequently change the *in situ* rheological properties. The low sensitivity of samples to cellulases with and without xyloglucanase supports the idea that readily enzyme-accessible xyloglucan does not play major roles in the mechanical properties of cell walls (Park and Cosgrove, 2012).

Although xyloglucan side-chains play a role in cell wall mechanical properties, their precise function remains an open question (Burgert and Dunlop, 2011; Park and Cosgrove, 2015). Test sets containing fucosidase demonstrated a low but significantly higher impact on *E* compared to the other test sets. The enzyme was inactive on the fucose containing a pectic rhamnogalacturonan II domain (data not shown) and was expected to primarily affect the last residue in the xylose-galactose-fucose xyloglucan side-chain. In combination with β-galactosidase, thought to further affect such branching, the impact on mechanical properties was enhanced. Alone, galactosidase had no effect on viscoelasticity (Fig. 2), probably due to the concurrent action of the endogenous activity of this enzyme in ripening apple (Goulao *et al.*, 2007; Wei *et al.*, 2010). Fucose on xyloglucan is a determinant for cell wall elongation (York *et al.*, 1984; Aldington *et al.*, 1991; Paque *et al.*, 2014) and the present results support its role in the mechanical properties of the cell wall. Details of its function remain to be elucidated with regard to xyloglucan/cellulose interactions (Whitney *et al.*, 2006) and/or in controlling the XTH remodeling of xyloglucan (Eklöf and Brumer, 2010; Maris *et al.*, 2011).

## Conclusions

The results of the present study revealed a complex relationship between the contributions of all cell wall polysaccharides to the cell wall mechanical properties, without their being specific associations with either elasticity or viscosity. The sensitivity of mechanical properties to enzymatic hydrolysis highlighted three key polysaccharide structures: methyl esterified HG, crystalline cellulose, and fucose on XyG side chains. Although degradation of HG probably induced cell–cell de-bonding with a major impact on tissue viscoelastic properties, the unexpected sensitivity of the mechanical properties of the cell wall to cellobiohydrolase and fucosidase points to an as yet unclear key function of the low amount of crystalline cellulose in the viscoelastic properties of fleshy fruit, and calls into question the regulatory role of the remodeling and/or metabolism of xyloglucan side-chains and its consequences on the mechanical properties of the tissue.

## Supplementary Data

Supplementary data are available at *JXB* online.

Modeling. Details of the mathematical models referred to in the text.

Fig. S1. Macroscopic image of cortex parenchyma tissue of Golden delicious after vacuum-infusion of the isotonic buffer.

Table S1. Relationship between the storage modulus and damping values.

Table S2. Sensitivity of the storage modulus variation to enzyme hydrolysis.

Table S3. Sensitivity of the relative variation of damping to enzyme hydrolysis.

## Supplementary Material

Supplementary_Model_Figure_S1_Tables-S1-S3
