# Supplementary material for: Examining the contribution of cell wall polysaccharides to the mechanical properties of apple parenchyma tissue using exogenous enzymes
Source: J Exp Bot. 2017 Sep 23;68(18):5137–46. doi: 10.1093/jxb/erx329 (PMC5853499; doi:10.1093/jxb/erx329)
Supplement: Supplementary_Model_Figure_S1_Tables-S1-S3 [file erx329_suppl_supplementary_model_figure_s1_tables-s1-s3.pdf]

## Supplementary data

### Modeling

### Constitutive equations of sample under uni-axial compression during enzyme hydrolysis

During enzyme hydrolysis (noted *Enz* in equation 1), polysaccharides sub-units are detached (*F* as *free* in equation 1) from cell wall polysaccharides (*B* as *bound* in equation 1):

$$B \overset{Enz}{\rightleftarrows} F \qquad (1)$$

The internal energy $\psi$of sample is a function of extensities (Callen 1985, Cunat 2001): the stretch ratio ($\lambda_v=V_0\,\lambda=V_0(1+\varepsilon)$, Magnenet *et al.*, 2012) created by the mechanical loading, the entropy ($S$) and mole numbers of bound and free polysaccharides ($N_b$, $N_f$.)

$$\psi = \psi(\lambda_v, S, N_b, N_f) \qquad (2)$$

Constitutive equations are deduced from equation 2 by differentiation (see Barbacci *et al.*, 2013; Magnenet *et al.*, 2012 for more detailed explanations):

$$\begin{bmatrix} d\frac{\partial\psi}{\partial\lambda_v} \\ d\frac{\partial\psi}{\partial S} \\ d\frac{\partial\psi}{\partial N_b} \\ d\frac{\partial\psi}{\partial N_f} \end{bmatrix} = \begin{bmatrix} \frac{\partial^2\psi}{\partial\lambda_v\partial\lambda_v} & \cdots & \frac{\partial^2\psi}{\partial\lambda_v\partial N_f} \\ \vdots & \ddots & \vdots \\ \frac{\partial^2\psi}{\partial\lambda_v\partial N_f} & \cdots & \frac{\partial^2\psi}{\partial N_f\partial N_f} \end{bmatrix} \cdot \begin{bmatrix} d\lambda_v \\ dS \\ dN_b \\ dN_f \end{bmatrix} \qquad (3)$$

Tisza's matrix is positive definite. In the general case, the coefficient of Tisza's matrix are functions of the extensive variables. It  can be rewritten in the lighter form:

$$\begin{bmatrix} d\sigma \\ dT \\ d\mu_b \\ d\mu_f \end{bmatrix} = \begin{bmatrix} E & \alpha & B_b & B_f \\ \alpha & \beta & D_b & D_f \\ B_b & D_b & G_{bb} & G_{bf} \\ B_f & D_f & G_{bf} & G_{ff} \end{bmatrix} \cdot \begin{bmatrix} d(1+\epsilon)V_0 \\ dS \\ dN_b \\ dN_f \end{bmatrix} \qquad (4)$$

with $\sigma$ the Cauchy longitudinal stress and the $\epsilon$ longitudinal strain created during the mechanical assay, $T$ the temperature, $\mu_i$ chemical potentials. Affinity ($A = \mu_b - \mu_f$) and speed of reaction ($d\xi/dt = dN_f/dt = -dN_b/dt$) are introduced in equation (4) leading to the reduced form of constitutive equations:

$$\begin{bmatrix} \dot\sigma \\ \dot T \\ -\dot A \end{bmatrix} = \begin{bmatrix} E & \alpha & B \\ \alpha & \beta & D \\ B & D & G \end{bmatrix} \cdot \begin{bmatrix} V_0\dot\epsilon \\ \dot S \\ \dot\xi \end{bmatrix} \text{ with } \dot\xi = L.A \qquad (5)$$

### Relation between storage modulus and damping

During experiment, temperature of the room was kept constant at 17°C. Temperature

variation (dT/dt=0) and thermal dilation is then negligible (*α=0*). Solving equation 5 leads to express affinity and rate of entropy as:

$$A(t) = \frac{-\dot{\epsilon}EV_0+\dot{\sigma}}{B.L} \text{ and } \dot{S}(t) = \frac{D}{B.\beta}(\dot{\sigma} - EV_0\dot{\epsilon}) \quad (6)$$

The rheological equation describing the relation between Cauchy stress and strains during enzyme hydrolysis is then:

$$\frac{d}{dt}\left(\frac{\dot{\sigma}-EV_0\dot{\epsilon}}{BL}\right) + (\dot{\sigma} - EV_0\dot{\epsilon})\left(\frac{G}{B} + \frac{D^2}{\beta B}\right) + BV_0\dot{\epsilon} = 0 \quad (7)$$

$\sigma$ and$\epsilon$ applied during DMA could be written:

$$\sigma(t) = \sigma_0 exp\big(i(\omega t + \delta)\big), \epsilon(t) = \epsilon_0 exp(i\omega t) \quad (8)$$

Injecting expression of complex stress and strain (equation 6) in equation 7 provides the storage modulus *E'* corresponding to the real part of the equation, *E''* the loss modulus the imaginary part:

$$E' = \frac{E\left(\beta G+D^2\right)^2 L^2 - \beta^2 B^2 L^2 (G-D^2)}{(BG+D^2)^2 L^2 + \beta^2\omega^2} \quad (9)$$

$$E'' = \frac{\beta^2 \omega B^2 L V_0}{(\beta^2 G^2 + 2\beta D^2 G + D^4)L^2 + \beta^2\omega^2} \quad (10)$$

Since $tan(\delta) = \frac{E''}{E'}$we obtained:

$$tan(\delta) = \frac{\beta^2 \omega B^2 L}{\left(E(\beta G+D^2)^2 - B^2\beta(\beta G - D^2)\right)L^2} \quad (11)$$

**Relation between storage modulus and tan(*δ*) for small damping values**

In the general case, *E'=E cos(δ).* If *δ<<1* then $E'=E+o(\delta^2)$. In this specific case the relation between damping and storage modulus (equation 11) can be written:

$$tan(\delta) = \frac{\alpha_1}{(\alpha_2 E' + \alpha_3)} \quad (12)$$

**Relation between E' and enzymatic hydrolysis**

Gibbs-Duhem relationships associated to the extensive variable *ψ* are (Callen 1985):

$$\begin{bmatrix} E & \alpha & B_b & B_f \\ \alpha & \beta & D_b & D_f \\ B_b & D_b & G_{bb} & G_{bf} \\ B_f & D_f & G_{bf} & G_{ff} \end{bmatrix} . \begin{bmatrix} V_0(1+\epsilon) \\ S \\ N_b \\ N_f \end{bmatrix} = 0 \quad (13)$$

We assumed the stiffness of the sample only in relation with bound polysaccharides ($B_f = 0$), entropy linked to free polysaccharides ($D_b = 0$) and no coupling effect between bound and free polysaccharides ($G_{bf} = 0$). Equation 13 becomes:

$$\begin{bmatrix} E & 0 & B_b & 0 \\ 0 & \beta & 0 & D_f \\ B_b & 0 & G_{bb} & 0 \\ 0 & D_f & 0 & G_{ff} \end{bmatrix} \cdot \begin{bmatrix} V_0(1+\epsilon) \\ S \\ N_b \\ N_f \end{bmatrix} = 0 \qquad (14)$$

Solving equation 14, to express E, β, $G_{bb}$, $G_{ff}$ leads to obtain:

$$E = \frac{-B_b N_b}{(\epsilon+1)V_0} \text{ with } N_b = N_b^0 - \xi = N_b^0 - \int_0^t \dot{\xi}\, d\tau \text{ and } E_0 = \frac{-B_b N_b^0}{V_0} \qquad (15)$$

**Sensitivity of storage and damping to enzyme hydrolysis**

Close to mechanical equilibrium ($\epsilon \approx 0$), assuming the speed of reaction constant ($\ddot{\xi} = 0 \Rightarrow \xi = \dot{\xi}t$) the variation of storage modulus is deduced from equation 15:

$$\Delta E' = E - E_0 = K_1 \dot{\xi} \Delta t \qquad (16)$$

The speed of reaction corresponds to enzyme activity, $\Delta t$ the time between infusion and testing. The sensitivity of storage modulus variation to hydrolysis is provided by the absolute value of the slope $K_1$.

Variation of damping (Δtan(δ)) is linked to the variation of storage modulus by equation 12:

$$\Delta E = \frac{-K_3 \Delta tan(\delta)}{tan(\delta) tan(\delta_0)} \qquad (17)$$

Equation 16 allows expressing the relative variation of damping as:

$$\frac{\Delta tan(\delta)}{tan(\delta)} = \frac{-K_1}{K_3} tan(\delta_0) \dot{\xi} \Delta t = K_2 \dot{\xi} \Delta t \qquad (18)$$

The sensitivity of the relative variation of damping to hydrolysis is then the absolute value of $K_2$.

*Additional references not cited in the main text*

Callen HB. 1985. Thermodynamics and an introduction to thermostatistics. New York: John Wiley & Sons.

Cunat C. 2001. DNLR approach and relaxation phenomena. Part I – Historical account and DNLR formalism. *Mechanics of Time-Dependent Materials* **5,** 39–65.

**Supplementary Figure S1:** Macroscopic observation of Golden cortex parenchyma tissue after vacuum infusion of the isotonic buffer. The vacuole in viable cells is stained red with 0.05% neutral red in isotonic buffer. No cell plasmolysis or bursting was observed. Air bubbles appear as black objects. Observed under Nikon AZ100 macroscope, lens 2x.

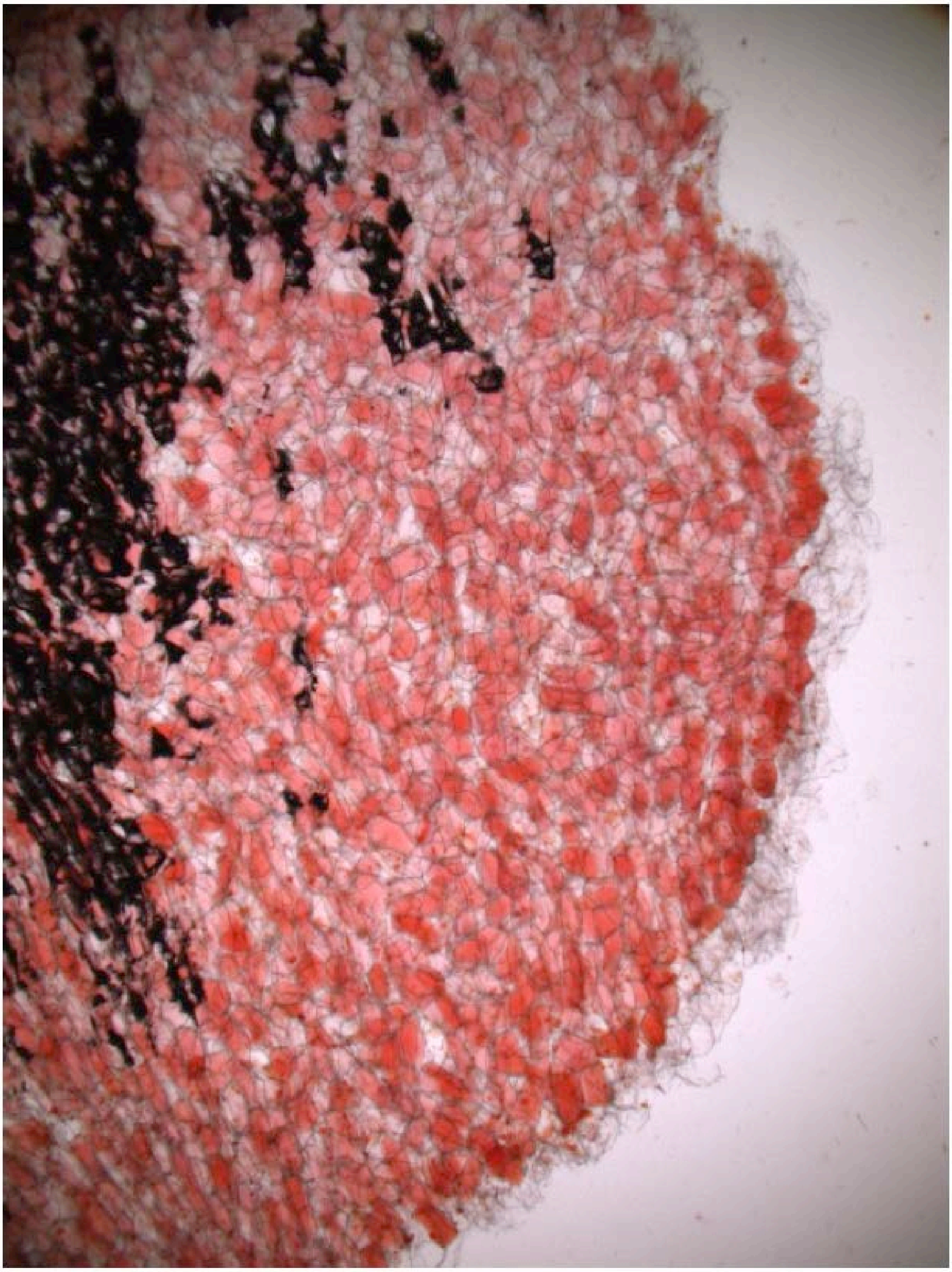

**Supplementary Table S1.** Relation between storage modulus and damping values

| Variety | Enzyme | Intercept | Intercept 95%LCI[a] | Intercept 95%UCI[a] | Slope | Slope 95%LCI[a] | Slope 95%UCI[a] | $R^2$ |
|---|---|---|---|---|---|---|---|---|
| **Go** | **af** | 2.46 | 2.13 | 2.80 | 1.99 | 1.69 | 2.29 | 0.81 |
| **Gr** | **af** | 2.03 | 1.60 | 2.46 | 1.68 | 1.37 | 1.99 | 0.52 |
| **Gr** | **afs** | 2.92 | 2.53 | 3.31 | 1.02 | 0.80 | 1.25 | 0.62 |
| **Go** | **afs+bgs** | 2.32 | 2.15 | 2.50 | 1.63 | 1.51 | 1.75 | 0.88 |
| **Gr** | **afs+bgs** | 3.58 | 3.32 | 3.84 | 0.68 | 0.50 | 0.85 | 0.50 |
| **Go** | **an** | 2.64 | 2.37 | 2.90 | 1.54 | 1.35 | 1.73 | 0.55 |
| **Gr** | **an** | 3.07 | 2.60 | 3.53 | 0.87 | 0.66 | 1.08 | 0.45 |
| **Gr** | **can** | 2.21 | 1.95 | 2.46 | 1.66 | 1.47 | 1.85 | 0.87 |
| **Go** | **can+xn** | 2.06 | 1.87 | 2.24 | 2.73 | 2.52 | 2.93 | 0.90 |
| **Gr** | **can+xn** | 2.28 | 2.07 | 2.48 | 1.78 | 1.60 | 1.96 | 0.83 |
| **Go** | **ch** | 2.37 | 2.19 | 2.55 | 2.00 | 1.85 | 2.15 | 0.85 |
| **Gr** | **ch** | 2.82 | 2.54 | 3.09 | 1.24 | 1.07 | 1.41 | 0.83 |
| **Go** | **ch+can** | 2.44 | 2.25 | 2.62 | 2.15 | 1.95 | 2.35 | 0.82 |
| **Gr** | **ch+can** | 1.87 | 1.60 | 2.14 | 2.44 | 2.13 | 2.76 | 0.85 |
| **Gr** | **ctl** | 3.53 | 2.88 | 4.18 | 0.77 | 0.47 | 1.08 | 0.24 |
| **Go** | **gn+an** | 2.79 | 2.45 | 3.12 | 1.56 | 1.30 | 1.82 | 0.76 |
| **Gr** | **gn+an** | 3.36 | 3.14 | 3.58 | 0.72 | 0.60 | 0.83 | 0.71 |
| **Go** | **pg** | 2.47 | 2.29 | 2.65 | 2.00 | 1.81 | 2.20 | 0.85 |
| **Gr** | **pg** | 1.92 | 1.43 | 2.41 | 1.60 | 1.33 | 1.87 | 0.72 |
| **Go** | **pl** | 2.11 | 1.91 | 2.31 | 1.98 | 1.82 | 2.15 | 0.84 |
| **Gr** | **pl** | 3.44 | 2.32 | 4.57 | 0.81 | 0.27 | 1.34 | 0.04 |
| **Gr** | **pme** | 2.16 | 1.11 | 3.21 | 1.19 | 0.82 | 1.56 | 0.23 |
| **Go** | **pme+pg** | 2.86 | 2.60 | 3.11 | 1.48 | 1.23 | 1.73 | 0.41 |
| **Gr** | **pme+pg** | 2.92 | 2.42 | 3.42 | 0.97 | 0.77 | 1.18 | 0.59 |

[a] Intercept 95%LCI, Intercept 95%UCI, Slope 95%LCI, Slope 95%UCI: Intercept and Slope 95% lower (L), upper (U) confidence interval

**Supplementary Table S2.** Sensitivity of variation of storage modulus to enzyme hydrolysis.

| Variety | Enzyme | Intercept | Intercept 95%LCI[a] | Intercept 95%UCI[a] | Slope | Slope 95%LCI[a] | Slope 95%UCI[a] | $R^2$ |
|---|---|---|---|---|---|---|---|---|
| **Go** | **af** | -2.65E-02 | -1.02E-01 | 4.86E-02 | -5.18E-06 | -6.05E-06 | -4.30E-06 | 0.54 |
| **Gr** | **af** | 3.89E-02 | -5.17E-02 | 1.30E-01 | -5.44E-06 | -6.51E-06 | -4.37E-06 | 0.46 |
| **Gr** | **afs** | 8.43E-02 | -1.21E-02 | 1.81E-01 | -1.57E-04 | -2.29E-04 | -8.48E-05 | 0.15 |
| **Go** | **afsbgs** | -2.20E-02 | -1.29E-01 | 8.53E-02 | -2.78E-04 | -3.59E-04 | -1.98E-04 | 0.27 |
| **Gr** | **afsbgs** | 5.98E-02 | -2.70E-03 | 1.22E-01 | -1.97E-04 | -2.43E-04 | -1.51E-04 | 0.40 |
| **Go** | **an** | 6.79E-02 | 2.56E-02 | 1.10E-01 | -7.77E-06 | -9.33E-06 | -6.21E-06 | 0.45 |
| **Gr** | **an** | 1.68E-01 | 9.27E-02 | 2.43E-01 | -1.73E-06 | -4.57E-06 | 1.11E-06 | 0.01 |
| **Gr** | **can** | 3.31E-02 | -7.38E-02 | 1.40E-01 | -1.58E-06 | -2.08E-06 | -1.08E-06 | 0.26 |
| **Go** | **canxn** | -1.04E-01 | -1.82E-01 | -2.62E-02 | -5.01E-06 | -6.09E-06 | -3.92E-06 | 0.44 |
| **Gr** | **canxn** | -1.04E-02 | -6.71E-02 | 4.64E-02 | -5.58E-06 | -6.37E-06 | -4.78E-06 | 0.62 |
| **Go** | **ch** | -2.03E-02 | -9.36E-02 | 5.30E-02 | -3.05E-03 | -3.74E-03 | -2.35E-03 | 0.40 |
| **Gr** | **ch** | -3.22E-03 | -8.31E-02 | 7.67E-02 | -2.40E-03 | -3.15E-03 | -1.65E-03 | 0.33 |
| **Go** | **chcan** | -5.72E-02 | -1.20E-01 | 5.60E-03 | -3.85E-03 | -4.42E-03 | -3.27E-03 | 0.61 |
| **Gr** | **chcan** | -4.81E-02 | -1.05E-01 | 8.74E-03 | -4.70E-03 | -5.24E-03 | -4.17E-03 | 0.84 |
| **Gr** | **ctl** | 1.47E-01 | 3.55E-02 | 2.58E-01 | -1.58E-06 | -2.10E-06 | -1.06E-06 | 0.23 |
| **Go** | **gnan** | -1.19E-02 | -8.89E-02 | 6.51E-02 | -1.28E-05 | -1.65E-05 | -9.16E-06 | 0.29 |
| **Gr** | **gnan** | 9.34E-02 | -9.69E-03 | 1.96E-01 | -7.29E-06 | -1.21E-05 | -2.50E-06 | 0.01 |
| **Go** | **pg** | -3.89E-02 | -1.22E-01 | 4.45E-02 | -6.62E-07 | -7.94E-07 | -5.30E-07 | 0.53 |
| **Gr** | **pg** | 1.30E-01 | 5.50E-02 | 2.05E-01 | -4.96E-07 | -6.13E-07 | -3.79E-07 | 0.38 |
| **Go** | **pl** | -1.16E-02 | -1.92E-01 | 1.68E-01 | -3.20E-02 | -3.89E-02 | -2.51E-02 | 0.52 |

| | | | | | | | | |
|---|---|---|---|---|---|---|---|---|
| **Gr** | **pl** | 1.40E-01 | -3.29E-03 | 2.82E-01 | -3.08E-02 | -3.61E-02 | -2.54E-02 | 0.60 |
| **Gr** | **pme** | 1.80E-01 | 1.16E-01 | 2.44E-01 | 4.99E-06 | 3.32E-06 | 6.66E-06 | 0.23 |
| **Go** | **pmepg** | 5.02E-02 | -1.71E-02 | 1.18E-01 | -1.43E-05 | -1.62E-05 | -1.23E-05 | 0.73 |
| **Gr** | **pmepg** | 2.30E-01 | 7.24E-02 | 3.88E-01 | -3.79E-06 | -8.34E-06 | 7.47E-07 | 0.03 |

[a] Intercept 95%LCI, Intercept 95%UCI, Slope 95%LCI, Slope 95%UCI: Intercept and Slope 95% lower (L), upper (U) confidence interval

**Supplementary Table S3.** Sensitivity of the relative variation of damping to enzyme hydrolysis

| Variety | Enzyme | Intercept | Intercept 95%LCI[a] | Intercept 95%UCI[a] | Slope | Slope 95%LCI[a] | Slope 95%UCI[a] | $R^2$ |
|---|---|---|---|---|---|---|---|---|
| **Go** | **af** | 0.01328 | -0.0396 | 0.06620 | 1.5e-06 | 9.2e-07 | 2.1e-06 | 0.21 |
| **Gr** | **af** | -0.02424 | -0.0540 | 0.00554 | 2.0e-06 | 1.7e-06 | 2.4e-06 | 0.52 |
| **Gr** | **afs** | -0.01757 | -0.0428 | 0.00763 | 8.0e-05 | 6.1e-05 | 9.8e-05 | 0.33 |
| **Go** | **afsbgs** | 0.01434 | -0.0358 | 0.06446 | 1.4e-04 | 1.0e-04 | 1.8e-04 | 0.22 |
| **Gr** | **afsbgs** | -0.02301 | -0.0406 | -0.00546 | 8.4e-05 | 7.1e-05 | 9.7e-05 | 0.61 |
| **Go** | **an** | -0.01690 | -0.0328 | -0.00099 | 3.1e-06 | 2.5e-06 | 3.6e-06 | 0.23 |
| **Gr** | **an** | -0.02527 | -0.0419 | -0.00867 | 1.2e-06 | 5.3e-07 | 1.8e-06 | 0.06 |
| **Gr** | **can** | -0.00462 | -0.0459 | 0.03668 | 5.8e-07 | 3.9e-07 | 7.7e-07 | 0.17 |
| **Go** | **canxn** | 0.02837 | -0.0044 | 0.06111 | 2.6e-06 | 2.1e-06 | 3.0e-06 | 0.35 |
| **Gr** | **canxn** | -0.01653 | -0.0380 | 0.00491 | 2.1e-06 | 1.8e-06 | 2.4e-06 | 0.45 |
| **Go** | **ch** | 0.01397 | -0.0210 | 0.04899 | 1.4e-03 | 1.1e-03 | 1.7e-03 | 0.20 |
| **Gr** | **ch** | -0.00058 | -0.0315 | 0.03034 | 8.7e-04 | 5.8e-04 | 1.2e-03 | 0.28 |
| **Go** | **chcan** | 0.00584 | -0.0242 | 0.03591 | 1.6e-03 | 1.4e-03 | 1.9e-03 | 0.41 |
| **Gr** | **chcan** | 0.00587 | -0.0217 | 0.03346 | 2.2e-03 | 2.0e-03 | 2.5e-03 | 0.64 |
| **Gr** | **ctl** | -0.03395 | -0.0541 | -0.01381 | 2.7e-07 | 1.7e-07 | 3.6e-07 | 0.13 |
| **Go** | **gnan** | 0.00455 | -0.0273 | 0.03636 | 4.7e-06 | 3.2e-06 | 6.2e-06 | 0.20 |
| **Gr** | **gnan** | -0.02026 | -0.0410 | 0.00047 | 3.4e-06 | 2.5e-06 | 4.4e-06 | 0.21 |
| **Go** | **pg** | 0.02106 | -0.0207 | 0.06283 | 2.6e-07 | 1.9e-07 | 3.2e-07 | 0.31 |
| **Gr** | **pg** | -0.01915 | -0.0408 | 0.00255 | 1.5e-07 | 1.1e-07 | 1.8e-07 | 0.17 |
| **Go** | **pl** | -0.03685 | -0.0816 | 0.00788 | 1.1e-02 | 9.7e-03 | 1.3e-02 | 0.56 |
| **Gr** | **pl** | -0.04196 | -0.0917 | 0.00775 | 2.6e-03 | 7.1e-04 | 4.4e-03 | 0.16 |
| **Gr** | **pme** | -0.02097 | -0.0402 | -0.00169 | 4.3e-07 | -7.6e-08 | 9.3e-07 | 0.01 |
| **Go** | **pmepg** | 0.00326 | -0.0164 | 0.02293 | 3.8e-06 | 3.3e-06 | 4.4e-06 | 0.56 |
| **Gr** | **pmepg** | -0.03666 | -0.0601 | -0.01317 | 8.0e-07 | 1.2e-07 | 1.5e-06 | 0.04 |

[a] Intercept 95%LCI, Intercept 95%UCI, Slope 95%LCI, Slope 95%UCI: Intercept and Slope 95% lower (L), upper (U) confidence interval
